# Logistic Model to Support Service Modularity for the Promotion of Reusability in a Web Objects-Enabled IoT Environment

**DOI:** 10.3390/s17102180

**Published:** 2017-09-22

**Authors:** Muhammad Golam Kibria, Sajjad Ali, Muhammad Aslam Jarwar, Sunil Kumar, Ilyoung Chong

**Affiliations:** Department of CICE, Hankuk University of Foreign Studies, Seoul 02450, Korea; kibria@hufs.ac.kr (M.G.K.); sajjad@hufs.ac.kr (S.A.); aslam.jarwar@hufs.ac.kr (M.A.J.); sunil75umar@hufs.ac.kr (S.K.)

**Keywords:** Internet of Things (IoT), Web of Objects (WoO), semantic ontology, object virtualization, reusability

## Abstract

Due to a very large number of connected virtual objects in the surrounding environment, intelligent service features in the Internet of Things requires the reuse of existing virtual objects and composite virtual objects. If a new virtual object is created for each new service request, then the number of virtual object would increase exponentially. The Web of Objects applies the principle of service modularity in terms of virtual objects and composite virtual objects. Service modularity is a key concept in the Web Objects-Enabled Internet of Things (IoT) environment which allows for the reuse of existing virtual objects and composite virtual objects in heterogeneous ontologies. In the case of similar service requests occurring at the same, or different locations, the already-instantiated virtual objects and their composites that exist in the same, or different ontologies can be reused. In this case, similar types of virtual objects and composite virtual objects are searched and matched. Their reuse avoids duplication under similar circumstances, and reduces the time it takes to search and instantiate them from their repositories, where similar functionalities are provided by similar types of virtual objects and their composites. Controlling and maintaining a virtual object means controlling and maintaining a real-world object in the real world. Even though the functional costs of virtual objects are just a fraction of those for deploying and maintaining real-world objects, this article focuses on reusing virtual objects and composite virtual objects, as well as discusses similarity matching of virtual objects and composite virtual objects. This article proposes a logistic model that supports service modularity for the promotion of reusability in the Web Objects-enabled IoT environment. Necessary functional components and a flowchart of an algorithm for reusing composite virtual objects are discussed. Also, to realize the service modularity, a use case scenario is studied and implemented.

## 1. Introduction

Recent technological advancement, a very large number of connected virtual objects, a significant amount of available data, and diverse service features enable the Internet of Things (IoT) infrastructure to create and offer services that facilitate society, economy, and daily living. Maintaining these very large numbers of connected virtual objects, as well as creating and offering intelligent services in the IoT environment is a complex task. Moreover, if new virtual objects need to be created for each new service, then a number of virtual objects will be increased exponentially, which burdens networks and system performance. Unavailable required virtual objects can be supported by other similar types of virtual objects. Hence, virtual objects need to be reused under similar circumstances that can provide similar functionalities.

Let us think of a scenario where a person moves to another city in order to attend a conference. During the conference, he stays in a hotel. He expects similar comfort (e.g., suitable temperature, humidity, light, etc.) in his hotel room as is in his home. An application system identifies the person, his current location at the hotel, and analyzes his personal profile and preferences. To create and offer user-preferred services, available real-world objects at the hotel room need to be controlled and managed as they are controlled and managed at the user’s home. Hence, service-relevant virtual objects and the composition of virtual objects are searched and matched for similar functionalities. Instead of creating new virtual objects and composing virtual objects to create and offer user-request services, the application reuses them as they were instantiated to offer services at home.

Service modularity is a key concept in a service-oriented architecture that allows for the reuse of existing virtual objects in heterogeneous ontologies. The reuse of existing virtual objects and their composites minimizes the complexity and the number of additional virtual objects needed under similar circumstances, avoids duplication, reduces the time it takes to search and instantiate them from their repositories, and increases the scalability and interoperability among multiple application domains. Considering these, the following factors have been identified:

- Service modularity in the Web of Objects (WoO) platform: WoO applies the principle of service modularity to create and offer IoT services by virtualizing real-world objects, composing them based on the service request, and reusing them in multiple application domains.
- Reuse of available virtual objects and composite virtual objects: To avoid complexity, ensure scalability, reduce the time it takes to search and instantiate similar virtual objects, etc., available virtual objects and composite virtual objects need to be reused. Cognitive functionalities can be applied to match similar virtual objects and composite virtual objects, or approximate them, which can provide similar functionalities under similar circumstances.

To overcome the lack of common standards of IoT at the application level, WoO [1,2] allows real-world object virtualization with the uses of semantic ontology, which forms a virtual object (VO) for information reusability, extendibility, and interoperability among multiple VOs. Each real-world object is represented by a virtual object, hence, controlling and managing a VO in the WoO platform means controlling and managing a real-world object in the real world. Real-world objects include physical objects and information in the real world. To offer intelligent services, composite virtual objects (CVOs) are created by combining multiple functionalities of VOs, constraints, and service policies. WoO allows the extension of the existing web with Web Objects in the IoT environment. In WoO, VOs are combined with web application characteristics, connected to the Internet, and applied to the web. WoO achieves distributed service infrastructure and adaptive service composition.

Service modularity is realized in terms of CVOs and their reusability in the WoO platform. Based on the user request, requested service-relevant CVOs and associated VOs are searched and matched. It is not necessary to have the CVO in its own application domain; if a CVO is not available in its own domain, it can be reused from another domain. Here, based on the user request, to create and offer requested services, the user and his current location are identified and service-relevant CVOs are instantiated. If the relevant CVOs are not available in their own ontology, then they are matched from other ontologies. Even though reusing real-world objects is important, and functional costs of reusing CVOs and VOs are just a fraction of deploying and maintaining real-world objects. Hence, reusing CVOs and VOs in the WoO platform is an important issue for resource optimization, information reusability, interoperability, knowledge-based service provisioning, etc. WoO is a service platform that deals with three levels for application-level service provisioning. This article focuses on reusing CVOs and VOs as well as their associated mechanisms.

Considering the abovementioned two factors, this paper proposes a logistic model that supports service modularity so that CVOs can be reused in multiple domains where similar types of VOs and their functionalities are available. Hence, this paper focuses on real-world object virtualization and its composition to support user requests, components in the WoO platform, and the reusability of CVOs.

In Reference [3], authors discussed the concept of VOs and proposed a log-based emergency management system. Authors presented a decision support tool to offer IoT services based on context information and a previous incident log. The authors claimed the use of a semantic ontology for reusability and interoperability. However, the key concept of reusability among multiple domains and its methodology have not been discussed in this article. Authors in Reference [4] proposed a semantic service composition architecture and presented a procedure for dynamic service composition. For user-centric dynamic service composition, the authors stated reusing and sharing of VO properties and attributes that were absent in the article. Compared to existing works, the main advantages of our proposed logistic model in the Web Objects-enabled IoT environment include the reusability of CVOs and VOs among multiple domains and real-world knowledge-based decisions, actions, and outcomes based on CVO reusability.

The structure of this paper is as follows: Section 2 presents the related research. Section 3 discusses features and functional components of the WoO platform and real-world object virtualization. Section 4 discusses the importance of reuse of CVOs and VOs. To reuse CVOs and VOs, this section presents and discusses the methodology of searching and matching them. This section also discusses the knowledge-based service provisioning in the WoO platform. Section 5 presents and implements a prototype for a use case scenario. This section performs experiments on the implemented functionalities and discusses the experiments. Finally, Section 6 concludes this article.

## 2. Related Works

Since IoT covers a vast area, a great deal of research has been conducted in this field. IoT promises a vision of connecting billions of objects using diverse communication technologies. At the same time, IoT enables companies and individuals to interact with these physical objects anytime and anyplace. IoT is a combination of different technologies, various protocols, and heterogeneous devices, altogether assuring the development of a system to provide smart services for end users. For several years IoT has become an active area in both academia and research. This was shown through the report “Cluster of European research projects on IoT” that was been presented in Reference [5]. The authors in this report discussed the vision and challenges for realizing IoT. Moreover, a cloud-centric vision for the implementation of IoT systems was deliberated in Reference [6]. The authors discussed important enabling technologies and applications which are expected to drive IoT research in the future. Further, they presented an implementation using the Aneka platform with private and public cloud interaction to support future IoT services. In Reference [7], the authors surveyed IoT and demonstrated the most important factors, including the communication solutions and integration of several other technologies. They discussed tracking and identification solutions in IoT, wired and wireless sensor networks and communication protocols, along with next generation Internet. Furthermore, they elaborated the importance of distributed intelligence in smart objects for IoT applications.

It is not always possible to connect real-world objects directly to applications. Hence, real-world objects, including physical objects and information, are virtualized to form VOs. A great deal of research has been done on object virtualization. VOs have become a major element in the IoT. A VO is the digital equivalent of a real-world object in IoT. VOs in IoT platforms help in the discovery and mashup of services and encourage the development of complex applications. In Reference [8], the authors surveyed VOs in the IoT world; discussed the definitions, characteristics, roles, and functionalities of Vos; and highlighted the common building blocks for the implementation of a virtualization layer. The authors also surveyed and presented the roles of VOs and the functionalities of architectures that have been implemented by different platforms, including oneM2M, SENSEI, IoT-A, COMPOSE, iCore, etc. Digital representations of real-world objects were discussed in Reference [9], where VOs carry application logic that enables them to sense the real world and interact with it. In Reference [9], the authors reported the design patterns for digital representations of VOs to identify the technical requirements for the future IoT. Moreover, in Reference [10], the authors demonstrated the IoT concept by describing how things in the real world integrate with the information technology virtual world. The authors in Reference [11] proposed an IoT prototyping toolkit (IoTLink). They demonstrated that their toolkit allows developers to create IoT mashup applications. Using visual components in the toolkit, it abstracts devices and services on the Internet as virtual entities, encapsulating the complexity of communication with them.

Object virtualization, harmonization, the composition of VOs and CVOs, and the WoO platform have been discussed and proposed in References [3,4,12,13,14,15]. A functional framework of WoO that includes the concept, reference model, functional capabilities, and information model were provided in the recommendation ITU-T Y.4452 [2]. Intelligent services can be created and offered by composing relevant VOs and CVOs, service logic, and constraints. Service composition requires synthesizing a specification to coordinate the components of service. Composition architecture, methodology, and algorithms for service composition were presented in References [16,17,18,19,20]. For reusability and interoperability, intercommunications among VOs is maintained using semantic ontology, which was discussed in References [21,22].

Discovering and combining relevant VOs for domain-specific service is possible for an application through cognitive functionalities. Cognitive functionalities enable CVOs to be self-managed, self-configured, and reused based on a service request. The concept of VOs, CVOs, and their functionalities were discussed in References [23,24,25].

A service is realized in terms of a CVO. To execute a service, the VOs that are relevant to the CVO need to be searched and matched in ontologies. In VO discovery, similarity between a source VO and a target VO is computed to match them. In an ontology, VOs and CVOs are defined in a hierarchical manner. Similarity between two VOs can be computed based on their hierarchical structure. Since VOs include different properties, to compute the similarity, all the properties of the source VO need to be compared with all the properties in the target VO. Structural matching combines different matching techniques [26] to compute the similarity between the properties of one ontology with the properties of another ontology. In this regard, two sets of synsets (sets of synonyms) of different properties can be computed using a Leacock Chodorow Matcher (LCM) [27,28].

Knowledge can be acquired and updated over the course of time using the concept of a hierarchy of classes, which is supported by the semantic ontology. Real-world information is captured, converted, and exploited to represent, organize, and reuse knowledge. Knowledge management processes and ontologies were discussed in References [29,30,31]. A knowledge creation mechanism was proposed and discussed in Reference [32].

Due to a rapid change of context, the application should have the ability to update itself with current context information. Context-awareness of real-world objects is necessary for any application for intelligent service provisioning. Context-awareness of objects was discussed in the BUTLER project and presented in Reference [33]. Context-aware services and applications were discussed in References [34,35].

## 3. Semantic Ontology Representation in the Web of Objects Platform

### 3.1. Web of Objects Archietcture

In the ubiquitous IoT environment, virtual objects interact with each other and share information to provide intelligent services, but they face isolation of information due to a lack of common standards. WoO facilitates the integration of isolated information from multiple application domains. Application deployment and operation are facilitated by the WoO platform. WoO allows the extension of the existing web with Web Objects in the virtual world.

**Figure 1:**
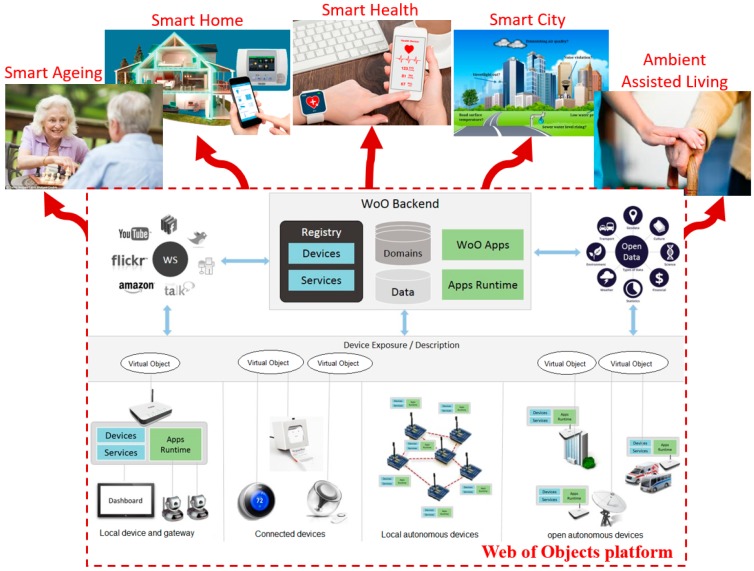
Example application domains in the Web of Objects platform [1].

Since real-world objects are not directly connected to applications, they are virtualized. WoO allows for the harmonization and composition of VOs to form application-level service features. With object virtualization, real-world objects can be monitored and controlled virtually as it is in the physical world. WoO is a service platform, where virtual space is created for the application domain, such as the smart home, smart city, smart health, etc., as shown in Figure 1.

**Figure 2:**
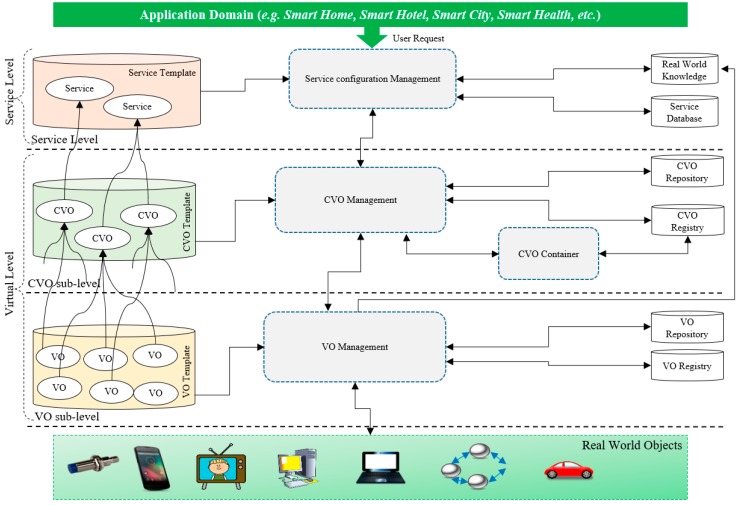
Functional entities and layer architecture of the Web of Objects platform [1,2].

The Web Objects-enabled IoT environment provides a simple approach that application developers and service providers develop and provide application features. The aggregation of VOs, CVOs, and service entities in the three-layer architecture of the WoO platform provides service features for end users and applications. The functional entities in the three-layer architecture of the WoO platform are shown in Figure 2.

In WoO, VOs perform an interface function to real-world objects. WoO allows VOs to represent functionalities and properties of real-world objects so that they can be accessed and used. Through sensing capabilities, meaningful information of real-world objects can be collected and stored at the VO level.

**Figure 3:**
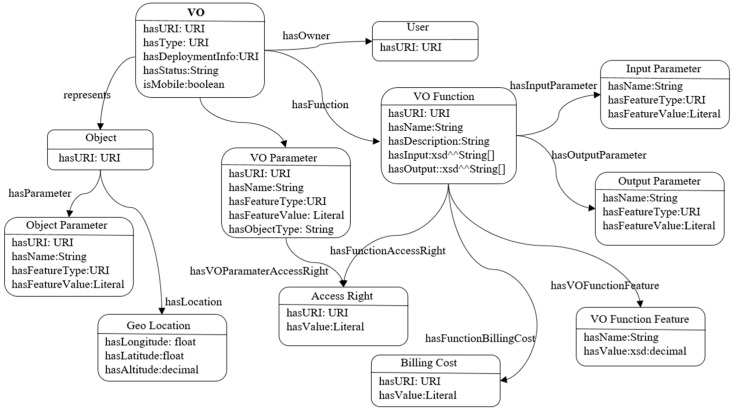
Virtual object (VO) information model.

Semantic ontology is used to describe a VO that can be accessed and shared semantically. In an application domain, the requested VO is instantiated from the VO template and stored in the VO repository. The VO template is created by the manufacturer and stored in the VO template repository. The VO is described in Resource Description Framework (RDF) format in order to be represented semantically. Metadata is represented in RDF and stored in an RDF graph database. The communications interface between the VO and real-world objects is done in a Representational state transfer (REST) or RESTful manner. For semantic interoperability among multiple VOs and a common understanding, a VO information model is used to describe a VO, which is shown in Figure 3.

In WoO, user-requested service is realized in terms of a CVO that is created by combining multiple VO functionalities, CVOs, and service rules. The CVO inherits all functions and features of the interrelated VOs and shares them with other CVOs that belong to internal and external domains. Available VO information and functionalities are retrieved from the VO repository for CVO creation.

**Figure 4:**
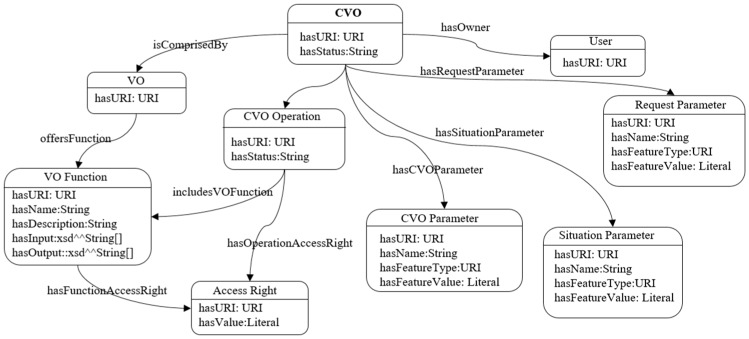
Composite virtual object (CVO) information model.

The CVO is instantiated from the CVO template and stored in the CVO repository. The CVO repository contains metadata including CVO identification, type, time of creation, validity, owner, CVO operation, access rights of the CVO, VO identification, etc. A CVO information model is used to describe a CVO, as shown in Figure 4.

A user-requested service is received at the service level, which performs as an interface between the user and WoO platform. A service is a logical mashup of CVOs, relevant VOs, and service policies. A service is not executed at the service level; rather, a service request is analyzed based on real-world knowledge, policies, and service request parameters that provide an appropriate service template to execute the service. The service level performs as a brain in WoO, thus, the performance of lower levels depends on the service level.

### 3.2. CVO Creation and Instantiation

A CVO template is used to instantiate a CVO to offer a user-requested service. A domain-specific CVO template is designed and created by a domain expert and stored in a CVO template repository. Different types of CVO templates are created in advance. The main goal of creating a CVO template is to reuse it if a similar type of CVO is requested under similar circumstances. Since a CVO is a combination of multiple VOs and/or CVOs and service rules, the information and functions of relevant VOs are retrieved to create a CVO template. An instantiated CVO is stored in a CVO repository, so, if the context and request parameters of a requested CVO match with the context and request parameters of a stored CVO, then the service might be created and offered directly.

**Figure 5:**
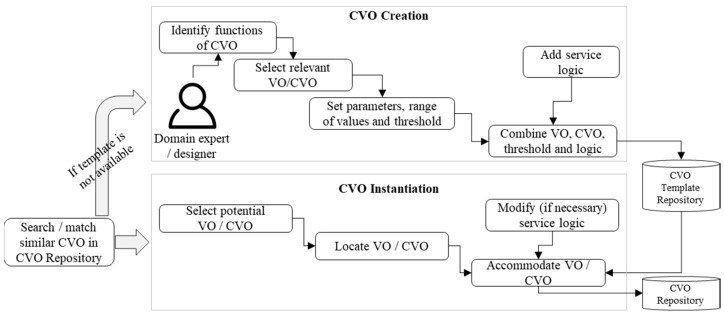
CVO creation and instantiation process at the CVO level.

Embedded cognitive functionalities in a CVO allow for the reuse of available CVOs and VOs. The functions and features of VOs and CVOs are combined in a CVO that can orchestrate with multiple other CVOs. A domain expert considers service requirements, service rules, context of a service, and available VOs to create a CVO. In a CVO creation and instantiation process, a requested CVO is searched and matched in a CVO repository based on the context and request parameters. If the requested CVO is matched, then the CVO is instantiated directly, otherwise the CVO is created. In the process of CVO creation, a designer first identifies what type of functions the CVO will provide. In the next step, the candidate VOs and CVOs are selected, and necessary parameters, ranges of the data values, and threshold values are set. Based on a service requirement, the selected VOs, CVOs, parameters, and service logic are combined and finally stored into a CVO template repository. In the process of a CVO instantiation, potential VOs and CVOs are selected and located. A CVO template is used to accommodate the selected VOs and CVOs. Depending on an application, a user-requested service might vary; hence, the service logic might be modified slightly for the instantiation process. Figure 5 shows a CVO creation and instantiation process.

## 4. Service Modularity for Reusability in the WoO Platform

### 4.1. CVO Reuse in the Web Objects-Enabled IoT Environment

If every time a new CVO needs to be created for each new service request, the complexity increases. To minimize the complexity, minimize the number of additional CVOs under similar circumstances, reduce the time it takes to instantiate them directly, and increase scalability and interoperability among multiple domains, CVOs need to be reused. The Web Objects-enabled IoT environment allows for the reuse of existing CVOs in multiple ontologies. Cognitive functionality at the CVO level, such as approximation and reuse functions, is used to search for similar CVOs to reuse them. Since completely identical CVOs are not available all the time, this function searches for similar or approximated CVOs that can support similar functionalities for current service requests within the current context. To approximate the CVO, the current context and request parameters are compared with the previous context and the request parameters of the CVO that was instantiated and stored in a CVO registry. The CVO information model includes metadata regarding context and request parameters. Context parameters include time, location, temperature, VOs, etc., whereas request parameters include VO function, policy, etc.

**Figure 6:**
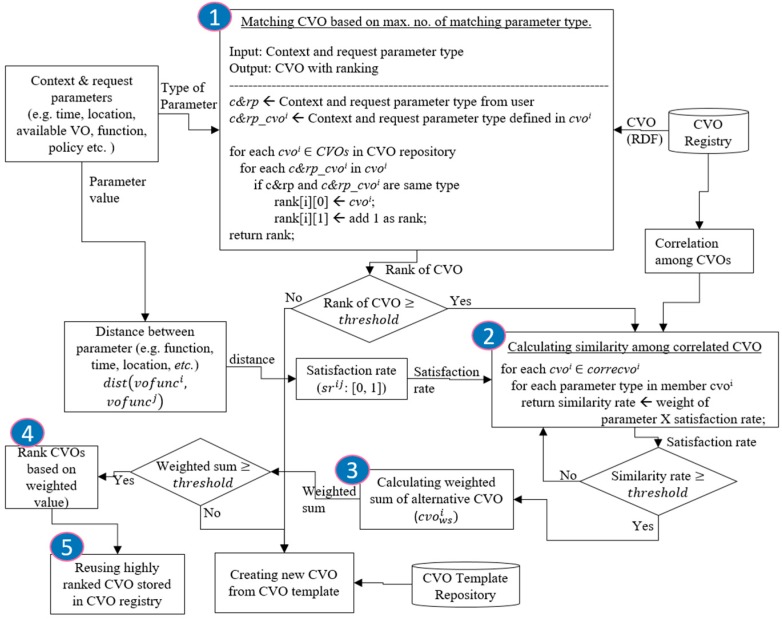
Flowchart of an algorithm for reusing CVOs.

Matching and comparing each and every CVO in the CVO registry is a complex and costly task. There should be a mechanism that allows matching and comparing within a limited number of CVOs or a group of correlated CVOs. A correlation matrix includes information regarding correlated CVOs that can be used to compare the limited number of CVOs. In this process, whenever a first matching CVO is found in the registry, a correlation matrix is consulted for correlated CVOs. A similarity value is calculated between each parameter in the correlated CVOs and, finally, a weighted sum [36] for the alternatively-correlated CVOs is calculated and compared against a threshold value. If the weighted sum is higher than, or equal to, the threshold value, then the CVO is ranked based on the weighted value. If none of the correlated CVOs’ weighted sums pass the threshold level, then the next CVO is searched, following a similar procedure until all the CVOs are searched in the CVO registry. If no CVO is matched or approximated, then the context and request parameters are forwarded to create a new CVO from scratch. A flowchart of an algorithm for reusing CVOs is shown in Figure 6.

Correlation among CVOs in the CVO registry is calculated, and the calculated values are stored in the correlation matrix. The calculation is done between requested functions and previous functions used in a previously-instantiated CVO. In the correlation matrix, 0 means there is no correlation between two functions and 1 means the requested function can be satisfied by the previously-used function; this implies that 0 means there is no correlation between CVOs and 1 implies that CVOs are highly correlated. Due to several reasons, such as changes in parameter types, the correlation among CVOs might be changed or invalid. Thus, a correlation matrix is validated continuously so that prediction accuracy is improved. If two CVOs have a high correlation among them, then it is normal that they perform similarly. Correlation between two CVOs can be defined in a range (0, 1). Correlation of CVOs can be expressed as:

(1) $$correlation\;\left(cvo^{i},\;cvo^{j}\right)=\{\begin{array}{c} 1,\;if\;\left|vofunc^{i},\;vofunc^{j}\right|\geq thres_{correlation}\\ 0,\;otherwise \end{array},$$

In Equation (1), *correlation* is calculated among $cvo^{i}$ and $cvo^{j}$ CVOs, where $vofunc^{i}$ and $vofunc^{j}$ are functions by *i*th and *j*th VOs, respectively, and $thres_{correlation}$ is the threshold value for correlation. If the range of the correlation among $vofunc^{i}$ and $vofunc^{j}$ is higher than, or equal to, the threshold value, then the condition is satisfied, and in that case (*i, j*) are highly-correlated CVOs.

A CVO can be highly correlated with multiple CVOs, where the CVO can perform as an active member of multiple correlated CVO pairs. In this case, $cvo^{i}$ is a member of a subset of correlated CVO pairs, $corrcvo^{i}$, that can be expressed as:

(2) $$cvo^{i}\in corrcvo^{i}=correlation\;\left(cvo^{i},\;cvo^{j}\right)=1,$$

If $vofunc^{i}$ is a set of functions in the requested CVO, and $vofunc^{j}$ is a set of functions in the stored CVOs, then a satisfaction rate is calculated to identify how close those functions are so that $vofunc^{i}$ is satisfied by $vofunc^{j}$ with respect to the correlated CVOs. The satisfaction rate $sr^{ij}$ between requested $vofunc^{i}$ and stored $vofunc^{j}$ function can be expressed as:

(3) $$sr^{ij}\;\left(vofunc^{i},\;vofunc^{j}\right)=1-\frac{dist\left(vofunc^{i},\;\;vofunc^{j}\right)}{\left|vofunc^{i}\right|}$$

In Equation (3), $dist\left(vofunc^{i},\;\;vofunc^{j}\right)$ is the distance between functions in the requested and stored CVOs that can be expressed in a range (0, 1). The distance between other features in the requested and stored CVOs can be calculated in a similar fashion.

Stated earlier, context parameters include time, location, available VOs, etc., and request parameters include functions and policies. These parameters are considered as criteria and defined in the CVO in the CVO registry. The satisfaction rate between the criteria stated in the requested and the stored CVO is calculated in terms of all of these features. Thus, overall similarity between requested and stored criteria can be calculated as the weighted sum of criteria, which can be expressed as:

(4) $$cvo_{ws}^{i}=\overset{n}{\underset{j=0}{\sum }}\left(wvofunc^{j}sr^{ij}+wcf^{j}\;sr^{ij}\right).$$

In Equation (4), $cvo_{ws}^{i}$ is an alternatively-correlated CVO specified in $corrcvo^{i}$ and *n* represents their relevant features. $wvofunc^{j}$ and $wcf^{j}$ are relative weights of VO function and context features, respectively, and $sr^{ij}$ is the satisfaction rate of these features in terms of $cvo_{ws}^{i}$. If the weighted value of $cvo_{ws}^{i}$ is higher than, or equal to, the threshold value, then the CVO is ranked; otherwise, the context and request parameters are forwarded to create a new CVO from scratch.

### 4.2. Discovery of a VO in an Ontology

A CVO is the combination of domain-specific multiple VOs. Based on the context and request parameters, a CVO is searched, matched, and ranked to be reused to offer a service. However, to execute the service, the CVO-relevant VOs need to be discovered and executed. Hence, the relevant VO names are extracted from a ranked CVO, and a VO discovery function is used to search and match them in the VO repository. Due to the very large number of available VOs, a reduction of the operational cost and an increase in the efficiency techniques need to be applied to limit the search space, such as using geographical location, time, etc. To discover CVO-relevant VOs, the properties of each source VO are matched with the properties of a targeted VO. Here, the source VO is the requested VO that needs to be searched and the targeted VO is the stored VO in the VO repository.

An ontology defines the VO and CVO in a hierarchal manner. For VO discovery, the similarity between a source and a target VO can be computed based on their hierarchical structure. In an ontology, a VO includes different properties including super-class, sub-class, object properties, data properties, domain, and range. To find the similarity, all the properties of a source VO need to be compared with all the properties of the target VO. Moreover, a higher number of properties matched between two VOs means the two VOs are more related to each other. The similarity result is the degree of matching; if the matching value exceeds the predefined threshold value, then the VOs are similar.

Let us consider a source VO $VO_{A}$ and a target VO $VO_{B}$ from two ontologies, *O_A_* and *O_B_*, that include the following features:

$$VO_{A}=\left\{Class_{VO_{A}},\;Properties_{VO_{A}}\right\},\;\mathrm{and}\;VO_{B}=\left\{Class_{VO_{B}},\;Properties_{VO_{B}}\right\}$$

where:

- $Class_{VO_{A}}$ is the super-class, sub-class, and associated classes of $VO_{A}$;
- $Class_{VO_{B}}$ is the super-class, sub-class, and associated classes of $VO_{B}$;
- $Properties_{VO_{A}}$ are the properties of $VO_{A}$;
- $Properties_{VO_{B}}$ are the properties of $VO_{B}$.

Similarity matching between $VO_{A}$ and $VO_{B}$ starts with computing the similarity between classes (e.g., super-class and sub-class) of $VO_{A}$ and $\;VO_{B}$. Later, the properties of the similar classes are retrieved and their similarities are computed. All the similarity values are summed to compute the similarity of $VO_{A}$ and $VO_{B}$. In general, the similarity between two VOs can be expressed as:

$$Similarity\left(VO_{A},\;VO_{B}\right)=\overset{n}{\underset{q=1}{\sum }}\overset{m}{\underset{p=1}{\sum }}\frac{Sim\left(Class_{pVO_{A}},\;Class_{qVO_{B}}\right)+Sim(Properties_{pVO_{A}},\;Properties_{qVO_{B}})}{({\left(NCl+NPr\right)}_{\;VO_{A}}+\left(NCl+NPr{)}_{VO_{B}}\right)/2}$$

where:

- ${\left(NCl+NPr\right)}_{\;VO_{A}}$ is the number of classes in $VO_{A}$ and properties in $VO_{A}$;
- ${\left(NCl+NPr\right)}_{\;VO_{B}}$ is the total number of classes in $VO_{B}$ and properties in $VO_{B}$.

For the similarity of classes, the sum of all similarity values between super-classes, sub-classes, and associated classes is calculated, and the result is divided by half the number of classes of $VO_{A}$ and $VO_{B}$. The expression is as follows:

$$\begin{array}{c} Sim\left(Class_{pVO_{A}},\;Class_{qVO_{B}}\right)\\ =\overset{n}{\underset{q=1}{\sum }}\overset{m}{\underset{p=1}{\sum }}\frac{(Sim\left(Sup_{pVO_{A}},\;Sup_{qVO_{B}}\right)+Sim\left(Sub_{pVO_{A}},\;Sub_{qVO_{B}}\right)+\;Sim\left(Asso_{pVO_{A}},\;Asso_{qVO_{B}}\right)}{\left(N_{Cl_{VO_{A}}}+N_{Cl_{VO_{B}}}\right)/2} \end{array}$$

where:

- $Sup_{pVO_{A}}$ and $Sup_{pVO_{B}}$ are the super-classes of $VO_{A}$ and $VO_{B};$
- $Sub_{pVO_{A}}$ and $Sub_{pVO_{B}}$ are the sub-classes of $VO_{A}$ and $VO_{B};$
- $Asso_{pVO_{A}}$ and $Asso_{qVO_{B}}$ are the associated classes of $VO_{A}$ and $VO_{B};$
- $N_{Cl_{VO_{A}}}\;\mathrm{and}\;N_{Cl_{VO_{B}}}$ are the total number of related classes of $Class_{pVO_{A}}\;\mathrm{and}\;Class_{qVO_{B}}$.

At the next stage, all the properties that are related to similar classes are extracted and the similarity between the properties of $VO_{A}$ and $VO_{B}$ is computed, which can be expressed as follows:

$$Sim(Properties_{pVO_{A}},\;Properties_{qVO_{B}})=\;\overset{n}{\underset{q=1}{\sum }}\overset{m}{\underset{p=1}{\sum }}\frac{\begin{array}{c} (Sim\left(Dp_{pVO_{A}},\;Dp_{qVO_{B}}\right)+Sim\left(Op_{pVO_{A}},\;Op_{qVO_{B}}\right)+\\ Sim\left(Dom_{pVO_{A}},\;Dom_{pVO_{B}}\right)+Sim\left(Ran_{pVO_{A}},\;Ran_{pVO_{B}}\right)\; \end{array}}{\left(N_{ProCl}+N_{DomPro}+N_{RanPro}\right)/2}$$

where:

- $Dp_{pVO_{A}}$ and $Dp_{qVO_{B}}$ are the data properties of similar classes $Class_{pVO_{A}}\;\mathrm{and}\;Class_{qVO_{B}};$
- $Op_{pVO_{A}}$ and $Op_{qVO_{B}}$ are the object properties of similar classes $Class_{pVO_{A}}\;\mathrm{and}\;Class_{qVO_{B}};$
- $Dom_{pVO_{A}}$ and $Dom_{qVO_{B}}$ are the domains of the properties;
- $Ran_{pVO_{A}}$ and $Ran_{qVO_{B}}$ are the ranges of the properties;
- $N_{ProCl}$ is the number of properties of similar classes $Class_{pVO_{A}}\;\mathrm{and}\;Class_{qVO_{B}}$;
- $N_{DomPro}$ is the number of domains;
- $N_{RanPro}$ is the number of ranges.

If the properties of two VOs are similar, then the VOs are similar. Similarity between two sets of synsets (sets of synonyms) of different properties are computed using LCM. If the similarity value of two VOs exceeds the predefined threshold, then LCM returns equivalence; otherwise, it returns *Idk* or “I don’t know”. LCM takes two synsets as input and computes the similarity between them by computing the shortest path between the two synsets. If the path between the two synsets is shorter, they are more related. The pseudocode of the algorithm for the similarity matching between two VOs is shown in Algorithm 1.

**Algorithm 1.** Similarity Matching**Input:** Ontologies $O_{A}$ and $O_{B}$, threshold; **Output:** Similarity of VOs;1:**for** each VO in $O_{A}$ and $O_{B}$2:  (
$VO_{p},\;VO_{q}$) = $\left(VO_{p}\in O_{A}\right)$, and $\;\left(VO_{q}\in O_{B}\right)$3:  $N_{c}$ = Total number of classes of $VO_{p}\;\mathrm{and}\;VO_{q}$4:  $N_{p}$ = Number of properties of $VO_{p}\;\mathrm{and}\;VO_{q}$5:  $N_{d}$ = Number of domains of $VO_{p}\;\mathrm{and}\;VO_{q}$6:  $N_{r}$ = Number of ranges of $VO_{p}\;\mathrm{and}\;VO_{q}$7:  ($Sup_{iVO_{p}},\;Sup_{jVO_{q}}$) = $\left(Sup_{iVO_{p}}\in VO_{p}\right)$, and $\left(Sup_{jVO_{q}}\in VO_{q}\right)$8:  ($Sub_{iVO_{p}},\;Sub_{jVO_{q}}$) = $\left(Sub_{iVO_{p}}\in VO_{p}\right)$, and $\left(Sub_{jVO_{q}}\in VO_{q}\right)$9:  ($Asso_{iVO_{p}},\;Asso_{jVO_{q}}$) = $\left(Asso_{iVO_{p}}\in VO_{p}\right)$, and $\left(Asso_{jVO_{q}}\in VO_{q}\right)$10:  **for** each $Sup_{iVO_{p}}$, $Sub_{iVO_{p}}$, $Asso_{iVO_{p}}$ in $VO_{p}$11:    **for** each $Sup_{jVO_{q}}$, $Sub_{jVO_{q}}$, $Asso_{jVO_{q}}$ in $VO_{q}$12:      // compute similarity using technique: LCM 13:      $S_{1}=Sim\left(Sup_{iVO_{p}},\;Sup_{jVO_{q}}\right)=LCM\;\left(Sup_{iVO_{p}},\;Sup_{jVO_{q}}\right)$14:      $S_{2}=Sim\left(Sub_{iVO_{p}},\;Sub_{jVO_{q}}\right)=LCM\;\left(Sub_{iVO_{p}},\;Sub_{jVO_{q}}\right)$15:      $S_{3}=Sim\left(Asso_{iVO_{p}},\;Asso_{jVO_{q}}\right)=LCM\;\left(Asso_{iVO_{p}},\;Asso_{jVO_{q}}\right)$16:   **end for**17:  **end for**18:  $Sim\left(Class_{VO_{p}},\;Class_{VO_{q}}\right)=\sum_{q=1}^{n}\sum_{p=1}^{m}\frac{S_{1}+\;S_{2}+\;S_{3}}{N_{c}/2}.$

19:  **for** each $Dp_{iVO_{p}}$, $Op_{iVO_{p}}$, $Dom_{iVO_{p}}$, $Ran_{iVO_{p}}$ in $Class_{VO_{p}}$20:    **for** each $Dp_{jVO_{q}}$, $Op_{jVO_{q}}$, $Dom_{jVO_{q}}$, $Ran_{jVO_{q}}$ in $Class_{VO_{q}}$21:     // compute similarity using LCM 22:     
$S_{4}=\;Sim\left(Dp_{iVO_{p}},\;Dp_{jVO_{q}}\right)=LCM\;\left(Dp_{iVO_{p}},\;Dp_{jVO_{q}}\right)\;$23:     $S_{5}=\;Sim\left(Op_{iVO_{p}},\;Op_{jVO_{q}}\right)=LCM\;\left(Op_{iVO_{p}},\;Op_{jVO_{q}}\right)$24:     $S_{6}=\;Sim\left(Dom_{iVO_{p}},\;Dom_{jVO_{q}}\right)=LCM\;\left(Dom_{iVO_{p}},\;Dom_{jVO_{q}}\right)$25:     $S_{7}=\;Sim\left(Ran_{iVO_{p}},\;Ran_{jVO_{q}}\right)=LCM\;\left(Ran_{iVO_{p}},\;Ran_{jVO_{q}}\right)$26:    **end for**27:  **end for**28:  $Sim\left(Prop_{VO_{p}},\;Prop_{VO_{q}}\right)=\sum_{q=1}^{n}\sum_{p=1}^{m}\frac{S_{4}+\;S_{5}+\;S_{6}+\;S_{7}}{\left(N_{p}+N_{d}+N_{r}\right)\;/\;2}.$

29:   **return**

30:  $Sim\left(VO_{p},\;VO_{q}\right)=\sum_{q=1}^{n}\sum_{p=1}^{m}\frac{\;Sim\left(Class_{VO_{p}},\;Class_{VO_{q}}\right)+\;Sim\left(Prop_{VO_{p}},\;Prop_{VO_{q}}\right)}{\left(N_{c}+N_{p}+N_{d}+N_{r}\right)/2}.$31: **end for**

### 4.3. Logistic Model to Support Service Modularity

To support the reusability of a CVO and VO in multiple application domains in the WoO platform for knowledge-based service provisioning, a logistic model has been proposed and instantiated. The sequence of operations starting from a user service request has been numbered. Initially, the user request service is taken to the service request analysis component to analyze the requested service. Context parameters from the knowledge database and service policies are also analyzed. Service request analysis provides the current context and request parameters to the service request execution component. Service request execution looks up service templates based on the parameters and forwards them along with the reference of service-relevant CVOs to the CVO management unit. Cognitive functionalities at the CVO level allow for the reuse of available CVOs. Functional components at the CVO sub-level search for similar types of CVOs in the CVO registry; if similar CVOs are matched, then they are instantiated directly to be executed; otherwise, the context and request parameters are forwarded to CVO creation to create a new CVO from scratch. The created CVO is registered in the CVO registry for future reuse. To execute a service, CVO-relevant VOs are also searched, matched, and reused.

**Figure 7:**
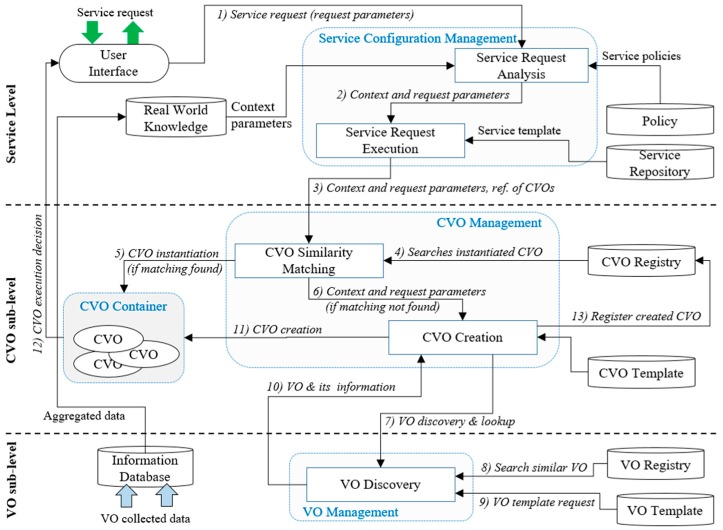
Logistic model to support service modularity in the Web of Objects (WoO) platform.

The recommendation ITU-T Y.4452 in Reference [2] provided a brief description of WoO functionalities. Based on the functionalities provided in the recommendation ITU-T Y.4452 and the functionalities that were proposed and implemented in References [3,4,17,18,23,32], the logistic model was proposed in the WoO platform, which includes several functionalities, in particular a CVO similarity matching and a VO discovery function to support service modularity. The logistic model is shown in Figure 7.

The CVO similarity matching functional component includes approximation and reuse functions that search instantiated CVOs in the CVO registry that can provide similar functions for the requested CVO. This function enables the reuse of matching CVOs, which minimizes service computation time and saves resources. This function evaluates available CVOs in the CVO registry. If a similar CVO is not matched in the CVO registry, then the CVO similarity matching component forwards the context and request parameters to the CVO creation component to create a CVO from a CVO template. To create a CVO from scratch, this function evaluates and selects the appropriate VOs to create an optimal CVO. The VO Discovery component discovers and looks up VOs in the VO registry and provides information of the requested VOs to the CVO Create component.

In general, initially, domain experts design a service template and define its parameters, but later the system dynamically modifies parameters based on knowledge. Knowledge is analyzed at the service level, but the process to create knowledge takes place at all levels in the WoO.

For reusability, prior knowledge regarding VOs and CVOs in the WoO platform is necessary. The main outcome of WoO is knowledge-based IoT service provisioning in heterogeneous ontologies. For knowledge-based intelligent service provisioning in the ubiquitous IoT environment, context-awareness of real-world objects has an important role.

In knowledge creation [32], context-awareness deals with linking changes in the ubiquitous IoT environment. Context-awareness enables the autonomous behavior of a system with minimal human involvement. Due to continuous changes of real-world objects in the IoT environment, real-world information should be processed for context-awareness. Context refers to a concept that characterizes and identifies associated entities, including users, location, time, objects, and information in a system. For context-aware services, applications are adapted to an environment by exchanging context information. Context-awareness enables an application to deduce context information in order to gain knowledge of users, location, time, services, etc.

Context-awareness is a core component of a knowledge-based system. Due to user mobility, service execution, etc., the context of real-world objects might be changed. Thus, knowledge needs to be updated. In WoO, for knowledge-based service provisioning, semantic ontology is used to accumulate, represent, and store knowledge. Hence, semantic ontology represents information in a structured manner, such as hierarchies of classes and sub-classes, where RDF is used to describe VO and CVO and is stored in Web Ontology Language (OWL) format. Raw data collected through a VO is stored in a database that is converted into meaningful information. Connecting and exchanging this information semantically forms knowledge.

**Figure 8:**
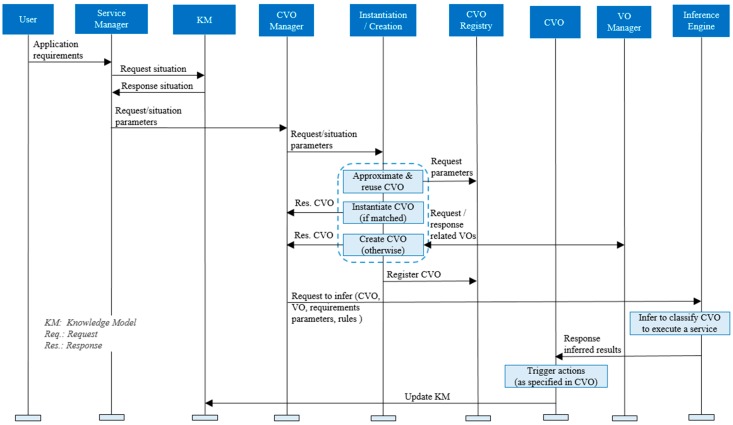
Sequence of instructions in the knowledge base for reusing a CVO in the WoO platform.

In WoO, knowledge is analyzed at the service level to select an appropriate service template that refers service-relevant CVOs and VOs. The service manager sends the request and situation parameters to the CVO manager to instantiate or create the CVO. The CVO manager searches the requested CVO in the CVO registry to reuse the CVO. If the requested CVO is available, then the CVO is directly instantiated, otherwise the CVO is created. To reuse a CVO, an approximation and reuse function is used to search for similar CVOs, which has been discussed earlier. Service-relevant VOs, constraints, threshold values, and policies are inferred to classify a CVO. If successfully classified, then predefined actions are triggered (e.g., sending a message to a smartphone). The sequence of instructions is shown in Figure 8.

## 5. Prototype Implementation for a Use Case in Smart Space

Smart space in the Web Objects-enabled IoT environment enables the creation of efficient space for various applications to create and offer intelligent services for users based on user identity, location, and activity. Personalized smart space in a web environment is required for seamless IoT service provisioning. Smart space in the Web Objects-enabled IoT environment includes different application domains and allows interrelation and interoperability among them, where a user-requested service might be provided by a domain that does not own, but reuses, required VOs and CVOs from other domains. Based on the service request, a query identifies the smart space service features in the home, office, crowded spaces, hospitals, hotels, etc. 

### 5.1. Use Case Scenario

A person has been visiting another city to attend a conference. During his stay in his hotel room, he expects similar comfort as is in his own home, such as suitable temperature, humidity, lighting, etc. As a user, he does not care about how the service will be created and offered, but expects that the service will be ready for him. He is satisfied in the sense that the hotel has maintained necessary safety measures to automatically identify emergency situations, such as fires, and to act on them.

### 5.2. Proof of Concept

From the use case scenario, two application domains, including the smart home and smart hotel, have been identified. The smart home provides a user-preferred room condition service that depends on the user’s health status and surrounding environment status. The smart hotel domain provides emergency services, such as identifying emergency situations and acting to prevent such situations. The smart hotel also provides guest health monitoring and environment monitoring services. 

In a smart home domain, userHealthMonitoring and environmentMonitoring CVOs have been instantiated to monitor the home user health status and home environment condition, respectively. Since the user-preferred room condition depends on the user’s health status and the surrounding environment, the userComfortMonitoring CVO has been instantiated, which combines userHealthMonitoring and environmentMonitoring CVOs.

In the smart hotel domain, the emergencySituation CVO has been instantiated to identify an emergency situation. guestHealthMonitoring and environmentMonitoring CVOs have been instantiated to monitor the guest’s health status and environment condition, respectively.

As stated earlier in the use case scenario, the guest expects similar comfort as is in his own home. For comfortable hotel room condition service, the required userComfortMonitoring CVO in the smart hotel domain is not available or defined. Since two other similar types of CVOs (guestHealthMonitoring and envStatusMonitoring CVO) are available in the smart hotel domain, userComfortMonitoring CVO from the smart home domain can be reused to create and offer service.

**Figure 9:**
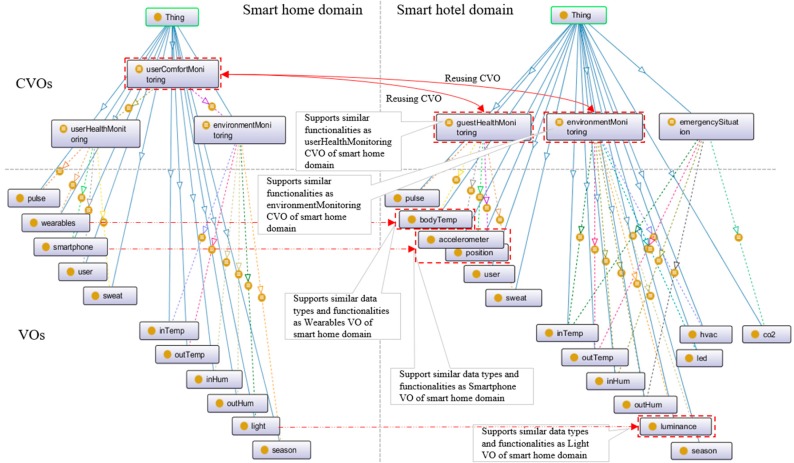
Similar types of VOs and CVOs in ontology models of use case scenarios.

Required VOs to represent relevant sensors and actuators in the smart home and smart hotel domains have been instantiated. Sensors include indoor and outdoor temperature sensors, a humidity sensor, a pulse sensor, a wearable sensor, a CO_2_ sensor, a light sensor, an accelerometer sensor, a position sensor, a body temperature sensor, a luminance sensor, a smartphone, etc. Actuators include light-emitting diode (LED), heating, ventilation, and air conditioning (HVAC), digital signage, alarms, fans, etc. Figure 9 shows two ontology models on two use case scenarios. Ontology models in the figure include VOs and CVOs, where the few of them that are similar have been marked using a red arrow. The figure shows that since four VOs and two CVOs in the smart hotel are found similar to those in the smart home domain, the guest request for similar service in the hotel room as provided in the smart home be created and offered by reusing the userComfortMonitoring CVO in the smart home domain.

### 5.3. Prototype Implementation

Based on the use case scenario, a prototype has been implemented. Implementation architecture includes an application server (AS), gateway, VO and CVO database, and a database to store data. Sensors, such as temperature, humidity, light, accelerometers, CO_2_, etc., and actuators, such as HVAC, LEDs, etc. have been connected through a gateway. All communications have been conducted through AS using RESTful manner. The AS runs the inference engine to deduce decisions based on available VO and CVO, threshold values, and currently-collected data, and instructs actuators to act.

In the implementation, TDB database has been used to store created VOs and CVOs. Due to its lightweight and high performance, TDB has been used to store and query RDF. To enable semantic web technology, the Apache Jena library has been used. The user can request service through a web interface using HTML5 and JavaScript. MongoDB has been used to store data. HTTP REST has been used for all sorts of communications. For interactive communications, such as sending and receiving data, WebSockets has been used.

A conceptual semantic ontology model on the use case scenario has been designed using an ontology editing tool called Protégé [37]. Required VOs and CVOs have been defined in RDF/ Extensible Markup Language (XML) format. The designed model has been represented in OWL and stored in an ontology database.

CVOs have been created by combining multiple relevant VOs and service rules. The ontology development tool, Protégé, allows an easy way to create a CVO by using its class expression editor. Sensor values have been set as a threshold during CVO creation, which has been inferred to make a decision.

In semantic ontology, service relevant VOs are interrelated, thus, necessary information can be extracted using a SPARQL Protocol and RDF Query Language (SPARQL) query. SPARQL query language has been used for querying the database in RDF. For querying the RDF, besides the prefix of the active ontology URI, universally-fixed prefixes, including rdf, owl, rdfs, and xsd, have been assigned.

Data collected through concerned VOs has been inferred to make an intelligent decision. The prototype was tested by running an inference engine through the AS using Hermit 1.3.7, which generated output values for CVOs. The inference engine ran on collected data, VOs, and rules against threshold values.

### 5.4. Results and Discussion

Specified features of the proposed logistic model have been verified and the performance of the implemented prototype has been evaluated. In the experiments, time and bytes that are required for executing components of the prototype have been considered. Experiments have been conducted against the number of VOs in the ontology. All the experiments have been repeated a number of times for the accuracy of the results, hence, mean values for each of the experiments have been used to generate the results.

**Figure 10:**
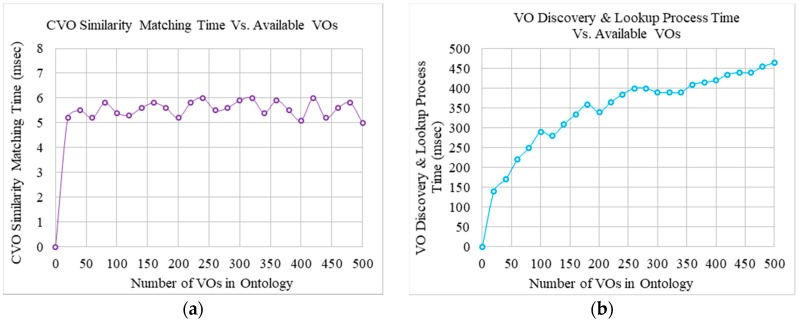
Observation of time required for executing (**a**) CVO similarity matching, and (**b**) VO discovery and search processes in terms of the increased number of VOs.

In the first experiment, the execution time for the CVO similarity matching component has been observed against the increased number of VOs. Results represented in Figure 10a show that the execution time for the CVO similarity matching remains steady, although the number of VOs increased. The results imply that the number of available VOs does not have any impact on this functional component.

The second experiments were conducted to observe the required time that is required to discover requested VOs and acquire their information from the VO registry with respect to the increased number of available VOs. The results that are shown in Figure 10b represent the time of the process that is required to discover and look up available VOs. The required time increases with respect to the increased number of available VOs, which is due to the creation time of the CVO, because a CVO is created by combining relevant multiple VOs and their functionalities. The figure shows that the average required time for the 100, 200, 300, and 500 VOs is 2.9, 1.7, 1.3, and 1.07 milliseconds, respectively. It is apparent that the discovery and search of the first 100 VOs takes the most of the total discovery and search time. The VO discovery and search time increases logarithmically rather than linearly for the later ones, due to the reuse of available VOs.

**Figure 11:**
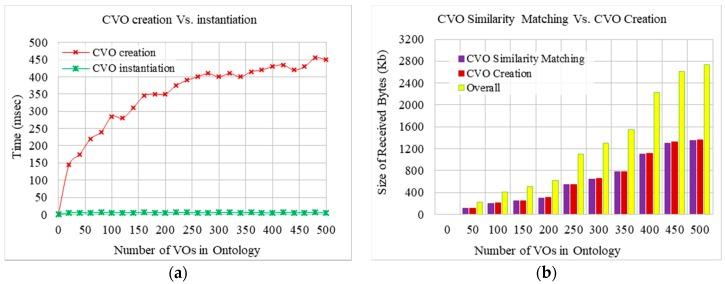
Comparison between (**a**) creation and instantiation process time, and (**b**) CVO creation and similarity matching components in terms of the required data.

Comparisons between CVO creation and instantiation process time with respect to the increased number of VOs was conducted in the third set of experiments. The results depicted in Figure 11a illustrate that the time required for CVO instantiation is lower than that for CVO creation. As discussed earlier, CVO instantiation only requires matching similar CVOs in the CVO registry, which depends on the similarity matching time, and does not require the discovery and search of relevant VOs and their information. On the other hand, CVO creation from scratch requires the discovery and search of requested VOs in the VO registry, and their composition requires additional time. Following the convention of Figure 10b, a similar trend can be visualized for a CVO creation while taking into account the VO discovery.

The comparison between CVO similarity matching and the CVO creation component in terms of received bytes was conducted and is shown in Figure 11b. The amount of received bytes represents the acquired information from the VO registry. As usual, the amount of received bytes increases due to the increased number of VOs, but the amount from both of these two components is similar because both of them acquire VO information for the requested VOs from the VO registry.

The experiments were conducted to validate the proposed logistic model in the WoO platform. The experiments show that the reusability of available CVOs and VOs reduces the creation and instantiation time and optimizes resources. Similar types of experiments were previously conducted in Reference [25]. Comparing to the research works performed in Reference [25], this article used a modular approach for the implementation of a CVO. Since a CVO is the combination of multiple CVOs and VOs, a service task was divided into multiple CVOs to support service modularity as a plug and play concept. To realize this service modularity, we implemented three different CVOs in the smart hotel ontology that could perform three different tasks and could be reused in other ontologies as well. Comparing to the experiments performed in Reference [25], our experiments performed better, as less execution time and resources were used for a greater number of available VOs.

## 6. Conclusions

Due to the immense number of virtual objects and the insufficient amount of available data, intelligent service provisioning in the IoT environment faces a lack of common standards. The Web Objects-based IoT environment addresses these lacking standards and facilitates application deployment and operations by virtualizing real-world objects. WoO allows the use of semantic ontology to virtualize real-world objects, where multiple VOs are combined for service provisioning. 

In the ubiquitous IoT environment, the number of virtual objects is increasing exponentially. Additionally, if a new virtual object needs to be created for each new service request, then they will burden the network and decrease system performance. WoO applies the principle of service modularity in terms of VO and CVO. Service modularity is a key concept in a service-oriented architecture that allows for the reuse of existing VOs and CVOs in the WoO platform. If a required VO and CVO are not available in a particular domain, then similar types of functionalities could be supported by similar types that are available in other domains. The reuse of existing VOs and CVOs allows for the reuse of similar types of functionalities, avoids additional and duplicate VOs and CVOs under similar circumstances, reduces the time it takes to search and instantiate directly from their repositories, and increases scalability and interoperability in heterogeneous ontologies.

This article proposes a logistic model that supports service modularity for the reuse of available CVOs and VOs in the Web of Objects platform. To support the reuse of CVOs and VOs, necessary functional components and methodologies to search and match CVO and VO discovery processes have been discussed. To realize the service modularity, a use case scenario has been studied and a prototype on the use case has been implemented.

To validate the proposed logistic model, experiments on the implemented functionalities have been performed. The experiments show that the reusability of available CVOs and VOs reduces creation and instantiation time and optimizes resources. Currently, we have been working on the implementation of identified functionalities. In our future work, we wish to evaluate performance in terms of capacity and scalability as a whole and provide a comprehensive analysis of this model.
